# Crystal structures of a natural DNA polymerase that functions as an XNA reverse transcriptase

**DOI:** 10.1093/nar/gkz513

**Published:** 2019-06-06

**Authors:** Lynnette N Jackson, Nicholas Chim, Changhua Shi, John C Chaput

**Affiliations:** 1 Departments of Pharmaceutical Sciences, University of California, Irvine, CA 92697-3958, USA; 2 Department of Chemistry, University of California, Irvine, CA 92697-3958, USA; 3 Department of Molecular Biology and Biochemistry, University of California, CA 92697-3958, USA

## Abstract

Replicative DNA polymerases are highly efficient enzymes that maintain stringent geometric control over shape and orientation of the template and incoming nucleoside triphosphate. In a surprising twist to this paradigm, a naturally occurring bacterial DNA polymerase I member isolated from *Geobacillus stearothermophilus* (Bst) exhibits an innate ability to reverse transcribe RNA and other synthetic congeners (XNAs) into DNA. This observation raises the interesting question of how a replicative DNA polymerase is able to recognize templates of diverse chemical composition. Here, we present crystal structures of natural Bst DNA polymerase that capture the post-translocated product of DNA synthesis on templates composed entirely of 2′-deoxy-2′-fluoro-β-d-arabino nucleic acid (FANA) and α-l-threofuranosyl nucleic acid (TNA). Analysis of the enzyme active site reveals the importance of structural plasticity as a possible mechanism for XNA-dependent DNA synthesis and provides insights into the construction of variants with improved activity.

## INTRODUCTION

Replicative DNA polymerases are highly faithful enzymes responsible for maintaining the genomic integrity of an organism (1). Structural insights into the mechanism of DNA synthesis have been obtained from crystal structures that capture the enzyme in different stages of nucleotide synthesis using non-reactive substrates or divalent metal ions that prevent phosphodiester bond formation (2–6). Bst DNA polymerase, a member of the DNA polymerase I family, has been extensively studied as a model system for DNA synthesis (7,8). Static structures depicting the four key mechanistic steps of the replication cycle (translocation, substrate binding, pre-catalysis, and post-catalysis) and their associated conformational changes are well documented (9). Together, these structures expand our understanding of the mechanism of DNA synthesis by illuminating the reaction pathway.

In addition to their natural activity, polymerases play an important role in many biotechnology applications, including next-generation DNA sequencing, molecular diagnostics, and therapeutics (10–12). Structure-function studies indicate that polymerases exhibit a broad tolerance for chemically modified 2′-deoxynucleoside triphosphates (dNTPs) that carry dyes, tags, or other organic groups at the C5 position of pyrimidines and the C7 position of 7-deazapurines (13). Crystal structures of thermostable DNA polymerases, like KlenTaq DNA polymerase (Klenow fragment of *Thermus aquaticus* DNA polymerase I, A-family) and the archaeal B-family polymerases of 9°N and Kod (*Thermococcus kodakarensis*), reveal that these bulky modifications pass through a large cavity that extends outside the enzyme active site (14–16). This cavity enables A- and B-family polymerases to incorporate C5-modified pyrimidines and C7-modified purines into the growing DNA strand and to continue synthesis after phosphodiester bond formation is complete.

By comparison, these same polymerase families are generally less tolerant toward chemical modifications made to the sugar moiety and often terminate primer extension when these analogs are present in the template or as incoming nucleoside triphosphates. This trend is especially true for xeno-nucleic acid (XNA) substrates in which the natural ribose and deoxyribose sugars found in RNA and DNA, respectively, have been replaced with a different sugar moiety (17). Prominent examples where sugar-modifications have been evaluated as templates for DNA synthesis include the recognition of: 2′,5′-isomeric DNA by the Klenow fragment of *Escherichia coli* DNA polymerase I (Kf exo-, A-family) and HIV reverse transcriptase (HIV RT, RT family) (18); locked nucleic acid (LNA) by Superscript III (RT family) (19); cyclohexenyl nucleic acid (CeNA) by HIV RT and the archaeal hyperthermophilc polymerase Vent (exo-, B-family) (20); hexose nucleic acid (HNA) by Kf (exo-) and the thermostable DNA polymerase I from *Thermus aquaticus* (Taq) (21); and α-l-threofuranosyl nucleic acid (TNA) by Superscript II and MMLV reverse transcriptase (RT family) (22). These efforts, though promising, were performed on templates that contain only limited numbers of modifications and often required increased time, elevated enzyme concentrations, or the presence of manganese ions to loosen the enzyme active site.

Efforts to overcome the stringent substrate specificity of natural polymerases against sugar-modified nucleotides led to the development of engineered polymerases that can copy genetic information back and forth between DNA and XNA (23,24). One exception to this rule is a naturally occurring bacterial DNA polymerase I member (A-family) isolated from the thermophilic species *Geobacillus stearothermophilus* (Bst), which exhibits innate reverse transcriptase activity on nucleic acid templates of diverse chemical composition, including a non-cognate RNA template (25), as well the synthetic congeners of glycerol nucleic acid (GNA) (26), 2′-deoxy-2′-fluoro-β-d-arabino nucleic acid (FANA) (27), and TNA (28). In the case of FANA and TNA, the reverse transcriptase activity was sufficient to enable the evolution of an RNA-cleaving FANA enzyme (FANAzyme) (27) and two examples of biologically stable TNA aptamers that bind to the protein targets of thrombin and HIV RT (29,30). To our knowledge, this is the only known example of a natural polymerase that can recognize FANA and TNA substrates by copying their templates into complete full-length DNA products. Motivated by the ability of Bst DNA polymerase to function as a general XNA reverse transcriptase, we sought to obtain an atomic level understanding of the molecular interactions that govern its recognition of non-cognate and synthetic congener templates.

Here, we describe two X-ray crystal structures of Bst DNA polymerase that capture the post-translocated product of DNA synthesis on templates composed entirely of FANA and TNA. These structures provide the first examples of an XNA reverse transcriptase and their bound substrates constitute the first structural view of chimeric DNA/FANA and DNA/TNA antiparallel Watson–Crick duplexes. Analysis of the enzyme active site provides insight into the mechanism by which a natural polymerase is able to recognize nucleic acid templates of diverse chemical composition and offers some rationale for how to design variants with improved functional activity.

## MATERIALS AND METHODS

### Oligonucleotide synthesis

FANA and TNA oligonucleotides were synthesized using standard phosphoramidite chemistry on an Applied Biosystems 3400 DNA synthesizer. TNA phosphoramidites were chemically synthesized while FANA phosphoramidites were purchased commercially (Glen Research). Both oligonucleotides were synthesized on 1 μmol scale Universal support™ 1000 solid support columns (Glen Research). The templates were cleaved from solid support and deprotected using 30% NH_4_OH at 55°C for 18 hours. The crude product from the TNA synthesis was purified using a Poly-Pak™ cartridge (Glen Research), followed by HPLC purification using a semi-preparative reverse-phase C18 150 × 4.6 mm column with 5 μm particle size with a 0–30% acetonitrile/ 0.1 M triethylammonium acetate (TEAA, pH 7.0) gradient elution. Finally, the TNA template was desalted using a Discovery^®^ C18 analytical 10 cm × 4.6 mm column with 5 μm particle size (Sigma Aldrich). The crude product from the FANA synthesis was purified using preparative denaturing polyacrylamide gel electrophoresis. Analytical HPLC was performed using a reverse-phase C18 150 × 4.6 mm column with 5 μm particle size to assess the purity of the FANA template. The identities of each template were confirmed using MALDI-TOF mass spectrometry.

### Cloning, expression and purification

The *Bst* (amino acid residues 299–876) gene was PCR amplified from a previously constructed pDEST007-*Bst* vector generously donated by Prof Thomas Carell (31) using Bst_for (ATC*CATATG*GCATTTACGCTTGCTGAC, IDT) and Bst_rev (ATGCGGC*GGTCTC*C TCGAGTCATTATTTCGCATCATACCACG, IDT) primers containing *NdeI* and *BsaI* restriction enzyme sites (underlined), respectively. Purified PCR product and the expression vector, pGDR11 (32), were digested with *NdeI* and *BsaI* restriction enzymes (NEB) and ligated and the resulting pGDR11-*Bst* construct was sequence verified (Retrogen). DH5-α cells (NEB) harboring pGDR11-*Bst* were grown aerobically at 37°C in LB medium containing 100 μg ml^−1^ ampicillin. At an OD_600_ of 0.8, expression of a tagless Bst was induced with 1 mM isopropyl β-d-thiogalactoside at 18°C for 16 h. Cells were harvested by centrifugation for 20 min at 3315 × g at 4°C and lysed in 40 ml lysis buffer (50 mM Tris–Cl pH 7.5, 1 mM EDTA, 10 mM BME, 0.1% v/v NP-40, 0.1% v/v Tween-20, 5 mg egg hen lysozyme) by sonication. The cell lysate was centrifuged at 23 708 × g for 30 min and the clarified supernatant was heat treated for 20 min at 60°C and centrifuged again at 23 708 × g for 30 min. The supernatant was loaded onto two 5 ml HiTrap Q HP columns (GE) assembled in tandem and washed with low salt buffer (50 mM Tris–Cl pH 7.5, 100 mM NaCl, 1 mM EDTA, 10 mM BME). Bst was eluted with a high salt buffer (50 mM Tris–Cl pH 7.5, 1M NaCl, 0.1 mM EDTA, 10 mM BME) using a linear gradient. Eluted fractions containing Bst were visualized by SDS-PAGE, pooled, and dialyzed against low salt buffer. The dialyzed sample was loaded onto a 5 ml HiTrap Heparin column (GE), washed with low salt buffer, and eluted using a linear gradient of high salt buffer. Eluted fractions containing Bst were visualized using SDS-PAGE and concentrated using a 30 kDa cutoff Amicon centrifugal filter (Millipore). Further purification was achieved by size exclusion chromatography (Superdex 200 HiLoad 16/600, GE) pre-equilibrated with Bst buffer (50 mM Tris–Cl pH 7.5, 150 mM NaCl, 1 mM EDTA, 10 mM BME). Purified Bst was concentrated to 20 mg ml^−1^ for crystallization trials using a 30 kDa cutoff Amicon centrifugal filter (Millipore).

### Reverse transcription procedures

DNA, RNA, FANA or TNA templates (5′-TCTCTATAGTGAGTCGTATAGGTGGTATCC-3′) and IR-700 labeled DNA primer (5′-GGATACCACC-3′) were chemically synthesized. The DNA template, DNA primer, and RNA template were ordered from Integrated DNA Technologies while FANA and TNA templates were synthesized using the methods above. The templates and primer underwent gel purification and electroelution prior to reverse transcription reactions. Each 10 μl reaction contained 10 pmol primer, 15 pmol template, 1× ThermoPol reaction buffer from New England Biolabs [20 mM Tris–HCl, 10 mM (NH_4_)_2_SO_4_), 10 mM KCl, 2 mM MgSO_4_, 0.1% Triton X-100, pH 8.8], 500 μM dNTP mix, 3 mM magnesium chloride and 4.5 μM Bst. The DNA reaction was incubated at 50°C for 1 h. The RNA and TNA reactions were incubated at 50°C overnight and the FANA reaction was incubated at 42°C overnight. Each reaction was quenched with 10 volumes of formamide buffer containing 25 mM EDTA.

### Crystallization procedures

#### General information

All reagents purchased from commercial suppliers were of analytical grade. Stock solutions of 2-methyl-2,4-pentanediol (Hampton Research), ammonium sulfate (Teknova), 2-(*N*-morpholino) ethanesulfonic acid (Calbiochem), magnesium chloride hexahydrate (Fisher Scientific) were filtered before use during crystallization trials.

#### Sample preparation

The templates (5′-GACGTACGTGATCGCA-3′, T) were synthesized and purified using the procedures above. The TNA and FANA templates have the same sequence, however, the FANA template contained uracil bases instead of thymine bases. The primer (5′-GCGATCACGT-3′, P), purchased from IDT, was used without further purification for crystallization trials. The P/T duplex was prepared by combining equal amounts of the primer and template strands in Bst buffer supplemented with 20 mM MgCl_2_, and annealing the strands by heating at 95°C for 5 min and cooling to 10°C over 10 min.

#### Crystallization

All polymerase samples were prepared at a final protein concentration of 4 mg ml^−1^. The samples were prepared by incubating Bst polymerase with 3 molar equivalents, for the FANA sample, and 1.5 molar equivalents, for the TNA sample, of the P/T duplex at 37°C for 30 min. Each sample had a second incubation with 10 M excess of dTTP and 10 mM manganese chloride at 37°C for 30 min to obtain the translocated structure. 24-well plate hanging drop trays were used to optimize crystal growth over a range of ammonium sulfate and MPD concentrations, based on previously published conditions (33) with each drop containing 1 μl of sample mixed with 1 μl of mother liquor over 500 μl of mother liquor in every well. Trays were stored in the dark at room temperature and crystal growth was generally observed after 2 days.

#### Data collection, structure determination and refinement

Diffraction datasets corresponding to the TNA and FANA structures were collected at the Advanced Light Source (Lawrence Berkeley National laboratory, Berkeley, CA) from single crystals. Images were indexed, integrated, and merged using XDS (34) and iMosflm (35). Data collection statistics are summarized in Table 1. Molecular replacement (MR) using Phaser (36) was performed for each of them. Initial data sets for both structures used the PDB structure 1L3S (5) as the search model, however, the published structures used refined models from previous data sets as MR search models. All final models were determined using iterative rounds of manual building through Coot (37) and refinement with phenix (38). The final stages of refinement employed TLS parameters for all structures. The stereochemistry and geometry of all structures were validated with Molprobity (39), with the final refinement parameters summarized in Table 1. Final coordinates and structure factors have been deposited in the Protein Data Bank. Molecular graphics were prepared with PyMOL and UCSF Chimera (40). 3DNA (41) and Tm-align (42) were used to analyze the structures.

**Table 1.** Crystallographic data collection and refinement statistics

	FANA	TNA
**Data Collection**		
Space group	*P*2_1_2_1_2_1_	*P*2_1_2_1_2_1_
Cell Dimensions		
* a, b, c* (Å)	87.8, 93.8, 105.8	87.3, 93.2, 103.7
α, β, γ (°)	90, 90, 90	90, 90, 90
Resolution (Å)	46.09–1.62 (1.67–1.62)	66.83–1.91 (1.98–1.91)
*R*_merge_	0.0411 (0.846)	0.7184 (2.131)
CC1/2	1 (0.824)	0.821 (0.588)
*I* / *σI*	23.32 (2.22)	41.14 (1.57)
Completeness (%)	99.73 (99.49)	98.70 (92.78)
Redundancy	6.6 (6.4)	19.17 (16.8)
**Refinement**		
Resolution (Å)	45.32–1.62 (1.67–1.62)	66.8–1.91 (1.98–1.91)
No. reflections	111376 (10941)	65329 (6039)
*R*_work_/*R*_free_	0.1819/0.2105 (0.255/0.2501)	0.193/0.225 (0.324/0.374)
No. atoms	5724	5433
Protein	4634	4585
Duplex	502	409
Solvent	637	338
B-factors		
Protein	37.37	43.09
Duplex	75.42	61.86
Solvent	45.23	43.69
R.m.s deviations		
Bond lengths (Å)	0.009	0.008
Bond angles (°)	1.20	0.80

*Values in parentheses are for the highest-resolution shell.

## RESULTS

### Unnatural reverse transcriptase activity

**Figure 1.**
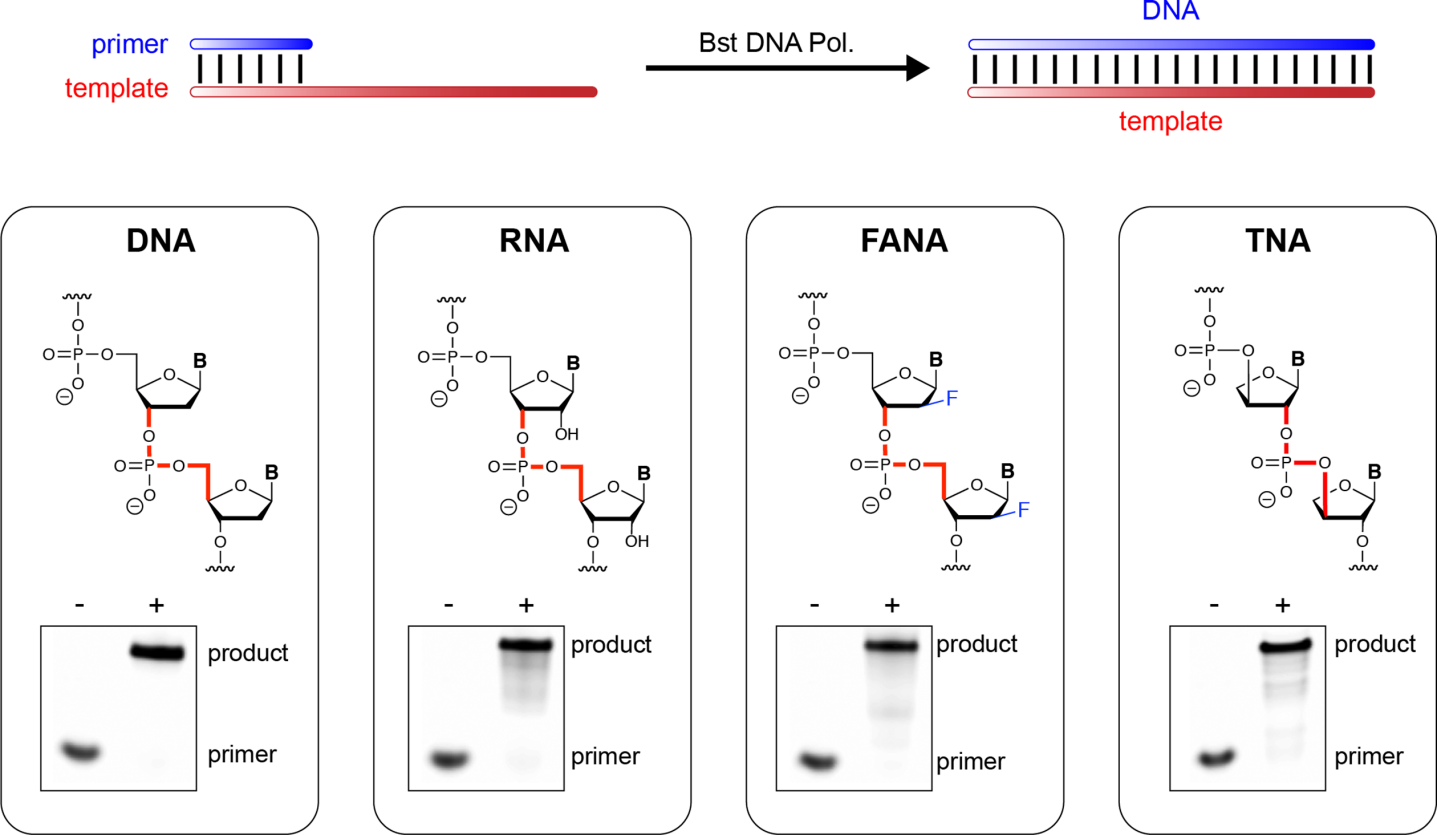
Polymerase-mediated DNA synthesis on diverse template compositions. Schematic illustration of DNA primer-extension on natural, non-cognate or synthetic templates. Insets show the molecular structures and polymerase activity for DNA synthesis on templates composed of DNA, RNA, FANA and TNA. Negative and positive symbols indicate the absence and presence of dNTP substrates.

We began by confirming the promiscuous activity of Bst DNA polymerase using a standard primer-extension assay. Accordingly, the polymerase was challenged to extend a primer-template (P/T) duplex in which the template strand was composed of entirely of DNA, RNA, FANA or TNA. The FANA and TNA templates used in this study were generated by solid-phase synthesis using commercial FANA phosphoramidites and chemically synthesized TNA phosphoramidites obtained in ∼15% overall yield from a 10-step chemical synthesis (43). Each duplex consisted of a 10 nucleotide (nt) primer-binding site followed by an unpaired 20 nt templating region designed to direct the synthesis of the complementary DNA strand (Figure 1). Analysis of the resulting primer-extension reactions by denaturing polyacrylamide gel electrophoresis (PAGE) demonstrates that natural Bst DNA polymerase is capable of catalyzing full-length DNA synthesis on natural (DNA), non-cognate (RNA), and synthetic congener (FANA and TNA) templates. This activity is consistent with previously published results and highlights the promiscuous nature of template recognition by a naturally occurring replicative DNA polymerase (25–28).

### Crystal structures of an XNA reverse transcriptase

**Figure 2.**
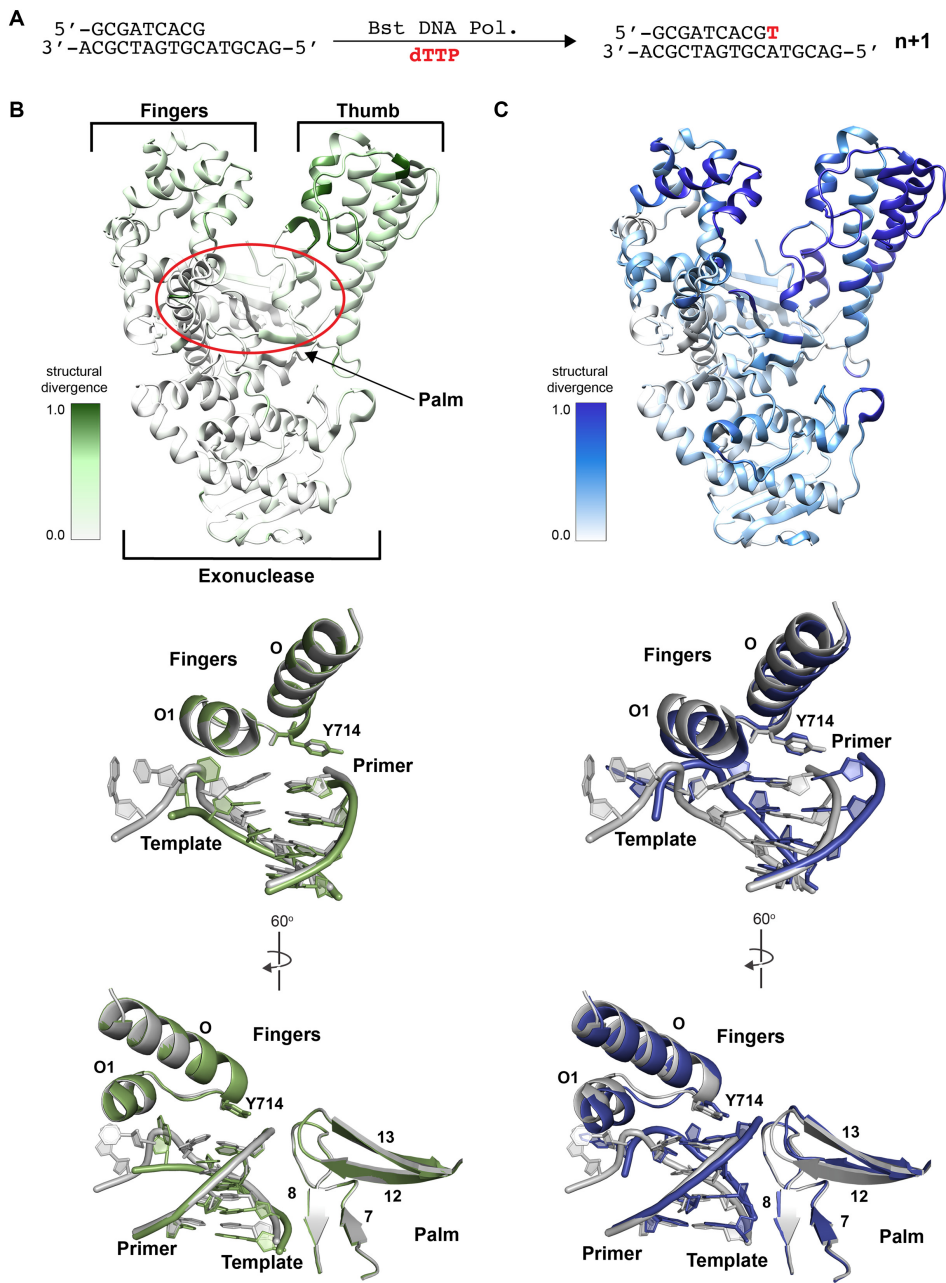
Crystal structures of post-translocated Bst DNA polymerase on XNA templates. (**A**) Schematic representation of the primer-extension assay used to generate the translocated product. (**B**, **C**) Structural analysis of Bst DNA polymerase following the addition of one DNA nucleotide to the 3′ end of a DNA primer annealed to either a FANA (B) or TNA (C) template. Heat maps (top) of Bst DNA polymerase reveal regions of structural divergence between the new structures and the natural system (PDB: 6DSY). The duplex was omitted for clarity. The active site (below) showing the fingers and palm regions of both structures (FANA, green and TNA, blue) superimposed against the natural system (gray).

Convinced that Bst DNA polymerase harbors innate reverse transcriptase activity, we chose to investigate the structural underpinnings of template recognition using X-ray crystallography. As an initial step in this direction, we pursued crystal structures of the translocated product of Bst DNA polymerase, which capture the product of a single DNA nucleotide addition onto the 3′ end of the DNA primer. For these experiments, we used the same P/T duplex (Figure 2A) described in previous studies so that the resulting structures could be compared with earlier structures obtained for the all-natural DNA P/T system (9). The only exception was the FANA template, which contained uridine residues in place of thymidine residues, as FANA thymidine amidites are not commercially available. Although crystallization studies were performed with RNA, FANA and TNA templates, suitably diffracting crystals were only obtained for the FANA and TNA systems. In these cases, co-crystals of the translocated product were acquired by crystallizing the product of a solution-catalyzed primer-extension reaction in which the polymerase and P/T duplex were incubated with TTP for 30 minutes at 37°C (Figure 2A). The crystal structures were solved to resolutions of 1.6 and 1.9 Å for the FANA and TNA bound structures, respectively (Table 1).

### Comparison of active site conformations with natural and XNA templates

The structures reveal a Y-shaped architecture common to A-family polymerases that encompasses the catalytic and exonuclease domains of the enzyme (Figure 2). The catalytic domain is further divided into the finger, palm, and thumb subdomains with the P/T duplex bound in a groove defined by the palm and thumb subdomains (Supplementary Figure S1). Superposition of the structures (Figure 2B, C) with a previously solved structure of Bst DNA polymerase bound to a fully natural DNA P/T duplex reveals subtle conformation changes in the finger, palm, and thumb subdomains (r.m.s. deviation for Cα atoms: 0.4 and 0.7 for the FANA and TNA containing structures, respectively). Structural differences of this type indicate that the polymerase engages the chimeric P/T duplex in slightly different conformations than is normally observed for a natural DNA duplex (9).

We evaluated the enzyme active site of both structures to identify molecular interactions that would allow Bst DNA polymerase to reverse transcribe XNA templates back into DNA. Relative to the natural system (9), the O and O1 helices of the finger subdomain and a loop connecting strands β12 and β13 of the palm subdomain change orientation to compensate for differences in the helical geometry of the chimeric heteroduplexes (Figure 2B, C). Structural deviations along the amide backbone are more pronounced for the TNA bound structure than the FANA bound structure, which is consistent with FANA being a close structural analog of DNA (44). Interestingly, Tyr^714^ maintains a similar orientation in both structures that stabilizes the newly formed base pair by stacking above the growing primer strand (Figure 2B, C). While this position in the DNA/FANA structure is isostructural to the natural system (9), it requires movement of the finger subdomain and loop connecting the O and O1 helices for Tyr^714^ to maintain proper stacking above the newly formed thymidine residue in the DNA/TNA structure. These observations reinforce the importance of Tyr^714^ as a critical checkpoint residue in the mechanism of DNA synthesis (45,46). The fact that Tyr^714^ is stacked above the added nucleotide in both structures implies that the mechanism of DNA synthesis is conserved for reverse transcription on XNA templates.

To maintain a conserved mechanism, protein contacts, particularly within the active site, are distinct (i.e. specific residues in the palm and finger subdomains form unique interactions to the P/T duplex). Interaction maps reveal that the chimeric duplexes are primarily recognized by contacts made to the phosphodiester backbone, with relatively few contacts being made to the sugar and nucleobase moieties (Supplementary Figures S2–S4). Several unique interactions are observed between active site residues, mostly from the palm subdomain, and the chimeric duplexes that are not observed in the natural system, including eight new interactions to the DNA/FANA duplex and 13 new interactions to the DNA/TNA duplex. Four of these contacts (Gly^590^, Asn^607^, Ser^617^ and Lys^434^) are observed in both structures. We postulate that these new molecular interactions are needed to position the template for DNA synthesis, as the local helical parameters differ from those observed for a standard DNA P/T duplex bound to Bst DNA polymerase (Supplementary Tables S1-S2).

### Helical parameters of chimeric DNA/XNA duplexes

**Figure 3.**
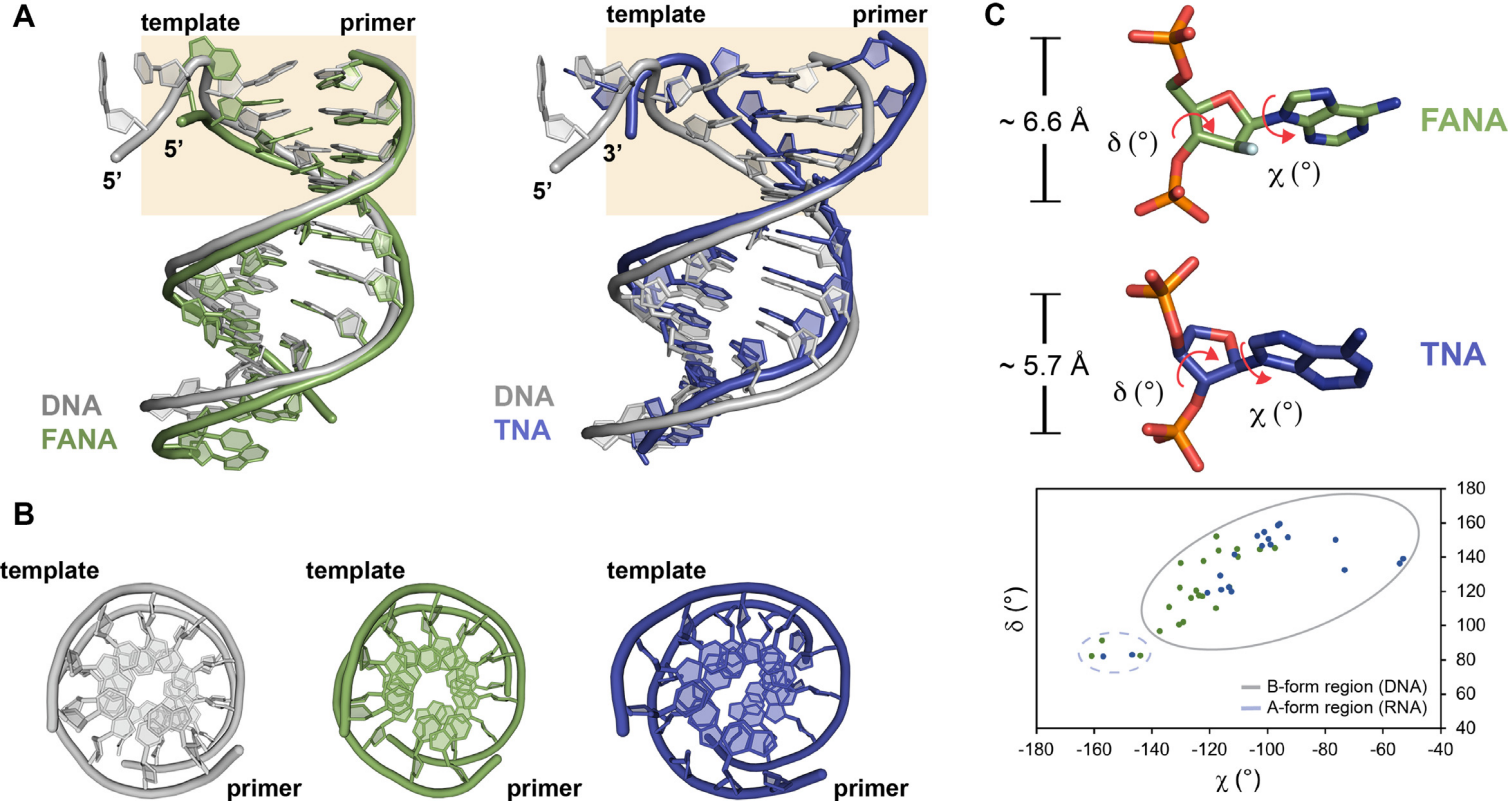
Structural analysis of the primer-template duplex. (**A**) Overlay of the DNA/FANA (green) and DNA/TNA (blue) primer-template duplexes with the DNA/DNA (gray) duplex from the natural system (PDB: 6DSY). The active site region is colored in wheat. (**B**) Top-down view from the active site reveals a preference for B-form helical geometries. (**C**) Representative nucleotides in the FANA and TNA templates show distinct sugar puckering and P···P distances. A covariance plot of χ, δ bond angles confirms that most nucleotides in the XNA templates (FANA, green and TNA, blue) favor a B-form helical geometry.

Despite differences in their helical geometry, both duplexes adopt B-form helical structures that are strikingly similar to the natural system (Figure 3A, B); the base pair inclination angles generally adopt B-form values over A-form values (Supplementary Table S1). This observation came as a surprise for TNA, which strongly favors an A-form helical geometry due to the rigid backbone formed by the trans-diaxial 2′,3′-phosphodiester linkages of α-L-threofuranosyl nucleotides (47,48). The clear preference for a B-form helix is presumably due to constraints imposed by the polymerase; however, such interpretations are speculative in the absence of solution structures of the free chimeric duplexes, which have not been solved for antiparallel DNA/FANA or DNA/TNA duplexes. Some insight into this problem was gained by comparing the backbone torsion angles (α, γ, δ, ϵ, ζ, noting that TNA lacks the β angle) for the DNA/TNA duplex versus a previous NMR structure of a TNA/TNA duplex (49), which reveals significant differences in the γ-angle (O3–C3 bond) along the duplex (Supplementary Table S3).

A residue-by-residue analysis reveals that threose favors a C3′-*endo* sugar conformation (Figure 3C, Supplementary Table S4), which differs from the C4′-*exo* conformation previously observed in known structures of TNA containing duplexes (49,50). By contrast, FANA maintains the same C1′-*exo* conformation observed in the solution structure of a FANA duplex (51). These observations are further supported by pseudorotation phase angles calculated for each duplex (DNA/TNA, DNA/FANA and DNA/DNA, Supplementary Figure S5). As expected, TNA exhibits shorter intranucleotide P···P distances (∼5.7 Å) than its complementary DNA strand (∼6.7 Å), which approximates values commonly observed for a standard B-form helix (Figure 3C, Supplementary Table S5) (52). The shorter P···P distance of the TNA strand is due to the backbone repeat unit of TNA, which has 5 atoms (or bonds) rather than the normal 6-atom backbone repeat unit found in DNA and RNA (53). Both strands of the DNA/FANA duplex have P···P distances of ∼6.6 Å, which closely approximates the all-natural DNA system (Figure 3C, Supplementary Table S5) (9).

**Figure 4.**
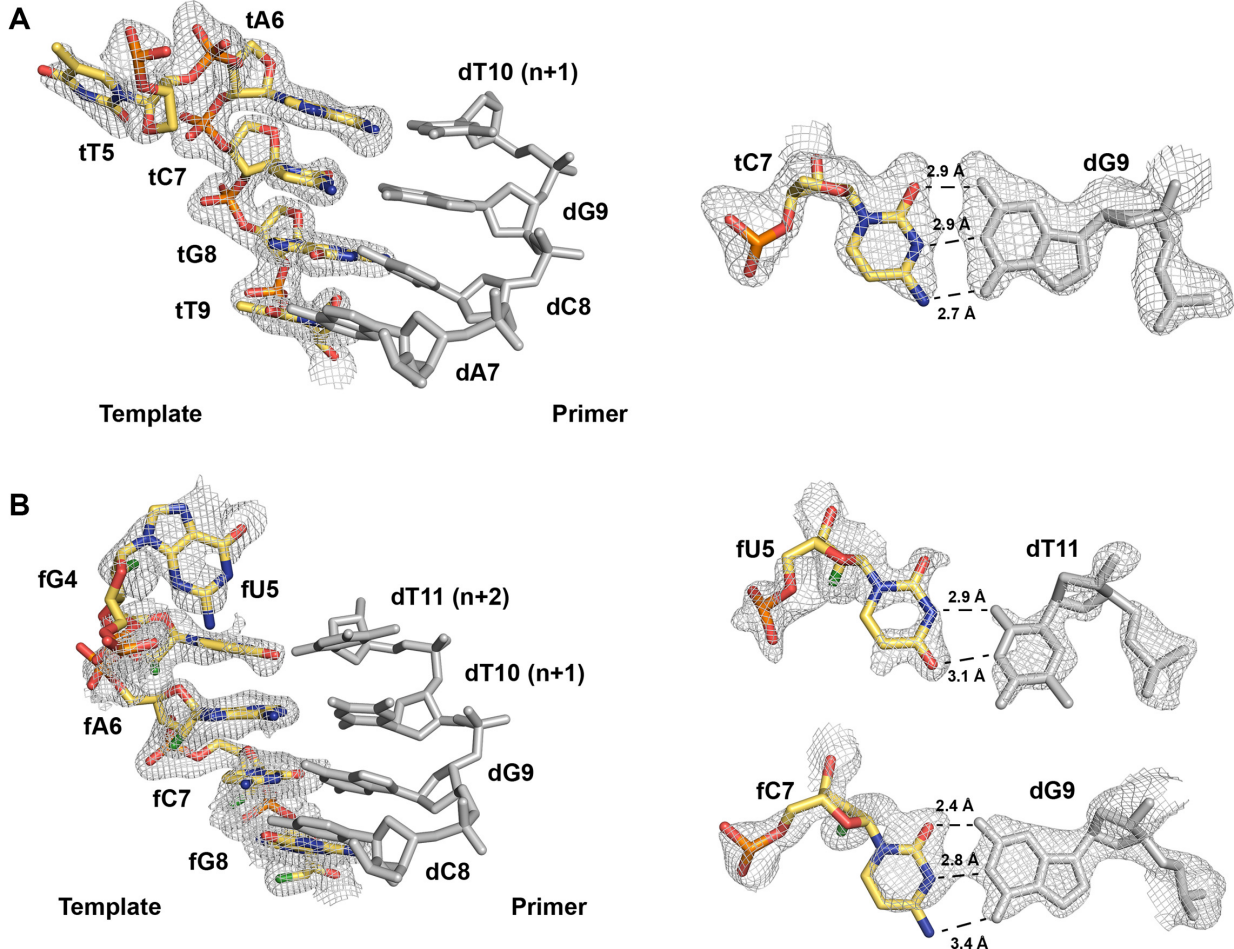
Heteroduplex active site omit maps. The active site encompassing the (**A**) DNA/TNA and (**B**) DNA/FANA heteroduplexes where simulated annealing Fo-Fc omit maps, contoured at 3σ and 2σ, are shown for the TNA and FANA templates, respectively. The template strands are colored yellow and the 2′-fluoro atoms in the FANA template are colored green. Shown to the right of the heteroduplexes are simulated annealing *F*_o_ – *F*_c_ omit maps, contoured at 2σ, for representative Watson–Crick base pairs for the heteroduplexes and the fU:dT mismatch in the FANA/DNA structure. Hydrogen bonds are depicted as black dashed lines with distances denoted for each base pair.

The high quality of the electron density maps provided a detailed view of the Watson-Crick base pairing interactions between the DNA/TNA and DNA/FANA chimeric duplexes (Figure 4). Despite having a backbone repeat unit that is one atom shorter than the complementary DNA strand, TNA forms standard Watson-Crick base pairs with DNA that are stacked at regular intervals of 3.4 Å distances. These interactions are due to the quasi-trans-diaxial orientation of the 3′ and 2′ positions, which helps maximize the separation distance between the 3′ and 2′ phosphate groups on adjacent nucleotides. Thus, although the distances between neighboring phosphates on the TNA strand is slightly less than the typical value expected for DNA (5.7 Å versus 6.6 Å), the conformation of the sugar compensates at a local level to facilitate base pairing with the DNA strand (50). By comparison, the DNA/FANA duplex is nearly identical to a standard DNA/DNA with a normal six-atom backbone repeat unit.

Close inspection of the translocated product indicates that Bst DNA polymerase successfully catalyzes the incorporation of a thymidine nucleotide opposite the templating adenosine residue in the TNA template (Figure 4A). This result is consistent with previous biochemical data showing that Bst can faithfully copy long strands of TNA templates into DNA (28). However, the post-translocated product observed in the FANA structure reveals formation of the *n* + 2 nucleotide addition product indicating that the polymerase underwent two rounds of catalysis. The first nucleotide addition generated the correct fA_6_-dT_10_ Watson–Crick base pair, while the subsequent addition produced an fU_5_-dT_11_ mispair at the *n* + 2 position (Figure 4B). Although pyrimidine-pyrimidine base pairing is rarely observed in structures (8), Benner *et al.* have shown that Watson-Crick duplexes composed entirely of pyrimidine-pyrimidine base pairs are possible (54). Relative to a previously solved Bst DNA polymerase structure for the natural *n* + 2 translocated product (9), the misincorporated dT_11_ nucleotide occupies the same position but exhibits greater rise, tilt, and twist than the correct dT_5_–dA_11_ Watson–Crick pair observed in the natural system (Supplementary Table S1). The second nucleotide adduct, which is presumably due to prolonged incubation of the enzyme with the TTP substrate, provides structural evidence for a fU–dT mispair, which could be relevant to future studies that examine the fidelity of DNA synthesis on XNA templates.

## DISCUSSION

Replicative DNA polymerases are responsible for maintaining the genomic integrity of their organism by accurately transmitting genetic information from one generation to another (55). Like most polymerases, the active site architecture is designed to rapidly incorporate DNA nucleotides into the growing strand by copying templates of diverse sequence composition (56). Active site residues are positioned to exclude RNA nucleotides (NTPs) that are present in the cellular milieu at much higher concentrations than the cognate dNTP substrates (57). Interestingly, many of the same interactions that allow DNA polymerases to discriminate against NTPs also provide strong discrimination against sugar-modified nucleotides that are present either in the template or as nucleoside triphosphates (58).

Bst DNA polymerase is unusual in that it exhibits a higher tolerance for templates containing sugar-modified nucleotides than is normally observed among naturally occurring replicative DNA polymerases. For example, other A-family members, like Taq DNA polymerase, which has been shown to copy RNA templates into DNA (59), is incapable of DNA synthesis on templates composed of either GNA or TNA (22,26). One possible explanation for the ability of Bst to function with reduced template specificity is the absence of 3′-5′ proofreading activity (60). Another possibility is that the enzyme active site is simply more flexible than other A-family polymerases and can readily adapt to the helical geometries of different chimeric duplexes.

**Figure 5.**
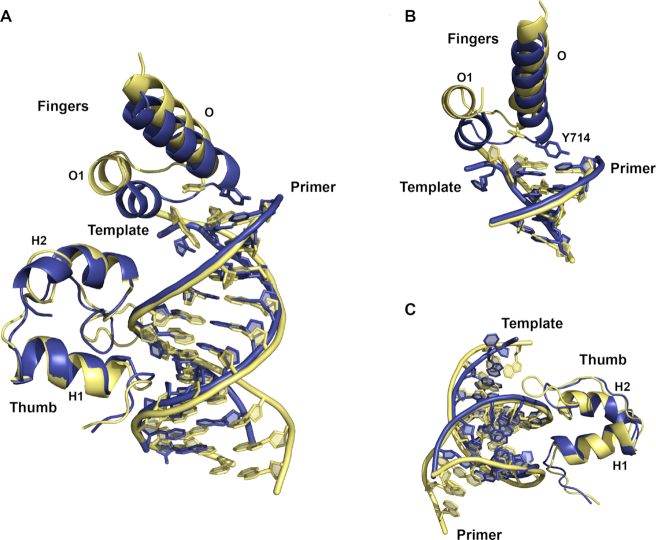
Superposition of Bst and KlenTaq DNA polymerases. The binary structure of Bst DNA polymerase bound to a DNA/TNA primer-template duplex (blue) was overlayed against the binary structure of KlenTaq DNA polymerase bound to an all-natural DNA primer–template duplex (yellow, PDB: 4KTQ). Structural views include the full duplex (**A**) and regions of the polymerase that exhibit conformational divergence (**B**, **C**).

However, it may be difficult to elucidate the precise rules that govern template recognition, as polymerases follow complicated reaction pathways that are still not fully understood. For example, previous structures of the post-translocated product of Bst DNA polymerase bound to an all-natural DNA P/T duplex have uncovered two different active site conformations (Supplementary Figure S6A) that appear to capture different steps in the reaction pathway (7,9). Superposition of the new TNA containing structure against both of the previous DNA containing structures (Supplementary Figure S6B, C) reveals that the TNA-containing structure is trapped in an active site conformation that is distinct from either of the two previous DNA-containing structures. In one case, the duplexes superimpose, while the finger subdomains are positioned in different locations (Supplementary Figure S6B). In the other case, the finger subdomains overlay, while P/T duplexes are located in different positions (Supplementary Figure S6C). A similar phenomenon is also observed when the TNA containing structure is superimposed onto an equivalent structure of KlenTaq DNA polymerase bound to an all-natural DNA P/T duplex (Figure 5) (61). In this case, the chimeric DNA/TNA duplex closely overlays with the natural DNA/DNA duplex, while the helices and loops in the finger and thumb subdomains occupy different conformations. Collectively, these structures highlight the complexity of polymerase-mediated DNA synthesis and the need for additional polymerase structures that probe the recognition of non-natural templates.

Some insights into the structural plasticity of Bst DNA polymerase were obtained by comparing the number of backbone interactions observed between side chain residues and the templating strand. The occurrence of such interactions increases from 16 to 21 when going from the DNA containing structure to the TNA containing structure (Supplementary Figures S2–S4). Notably, the number of interactions made to the primer strand remains roughly constant in both structures, suggesting that TNA reverse transcription is driven by template recognition. If we assume that the number of side chain contacts made to the template strand is an indication of enzyme flexibility, then the ability for Bst DNA polymerase to accommodate additional contacts beyond what is already observed in the natural system implies that Bst maintains a high degree of enzyme flexibility. Although Taq DNA polymerase adopts a similar topology, its inability to bind TNA templates in a productive conformation suggests that it is less flexible (22).

Ongoing efforts to establish XNA polymerases with improved reverse transcriptase activity should focus on specific residues in the active site of Bst DNA polymerase that form important interactions to the template strand. For example, Tyr^499^, Phe^781^ and Arg^729^, are logical starting points for rational design or site-directed mutagenesis, as these residues form contacts to the DNA template but not the FANA or TNA templates. However, if more significant changes are needed to achieve heightened activity, then all of the P/T contacts observed in the natural and XNA structures could be surveyed for improved activity using droplet-based microfluidic approaches that allow for high throughput screening in uniform microcompartments (23).

In the context of biotechnology, Bst DNA polymerase provides a powerful tool for reverse transcribing certain XNAs back into DNA where they can be read by conventional DNA sequencing technologies. The ability to exchange genetic information from one biopolymer system to another is critical for the in vitro selection of XNA affinity reagents and catalysts. Unlike DNA, XNA reagents offer increased stability against nucleases that degrade oligonucleotides, thereby providing a route to biologically stable reagents for diagnostic and therapeutic applications (62). As these studies advance, it will be interesting to see how many different XNA templates can be reverse transcribed using Bst DNA polymerase. Such experiments provide an opportunity to expand the chemical space of artificial genetic polymers that are capable of Darwinian evolution, which is a major goal of synthetic biology with future practical applications in medicine and biotechnology (63).

In summary, we describe high resolution crystal structures of Bst DNA polymerase that capture the translocated product of DNA synthesis on templates composed entirely of FANA and TNA nucleic acid polymers. Comparison of these structures against the natural system indicates that the enzyme active site is able to adapt to the helical geometry of the chimeric P/T duplex. We suggest that this property, in conjunction with directed evolution, could enable the evolution of highly specialized XNA polymerases for synthetic biology.

## DATA AVAILABILITY

Coordinates and structure factors have been deposited in the PDB with the accession codes: 6MU4 (Bst structure with FANA template) and 6MU5 (Bst structure with TNA template).

## Supplementary Material

gkz513_Supplemental_File
