# Supplementary material for: Crystal structures of a natural DNA polymerase that functions as an XNA reverse transcriptase
Source: Nucleic Acids Res. 2019 Jun 6;47(13):6973–83. doi: 10.1093/nar/gkz513 (PMC6649750; doi:10.1093/nar/gkz513)
Supplement: gkz513_Supplemental_File [file gkz513_supplemental_file.pdf]

Supporting Information

Crystal Structures of a Natural DNA Polymerase that Functions as an XNA Reverse Transcriptase

Lynnette N. Jackson[1#], Nicholas Chim[1#], Changhua Shi[1], and John C. Chaput[1,2,3]*

[1] Departments of Pharmaceutical Sciences, University of California, Irvine, CA 92697-3958.

[2] Department of Chemistry, University of California, Irvine, CA 92697-3958.

[3] Department of Molecular Biology and Biochemistry, University of California, CA 92697- 3958.

* To whom correspondence should be addressed. (e): jchaput@uci.edu, (t) 949-824-8149
[#] These authors contributed equally to the manuscript

* To whom correspondence should be addressed. (e): jchaput@uci.edu
[#] These authors contributed equally to the manuscript

Table of Contents

Supplementary Figures S1-S6
Supplementary Tables S1-S5

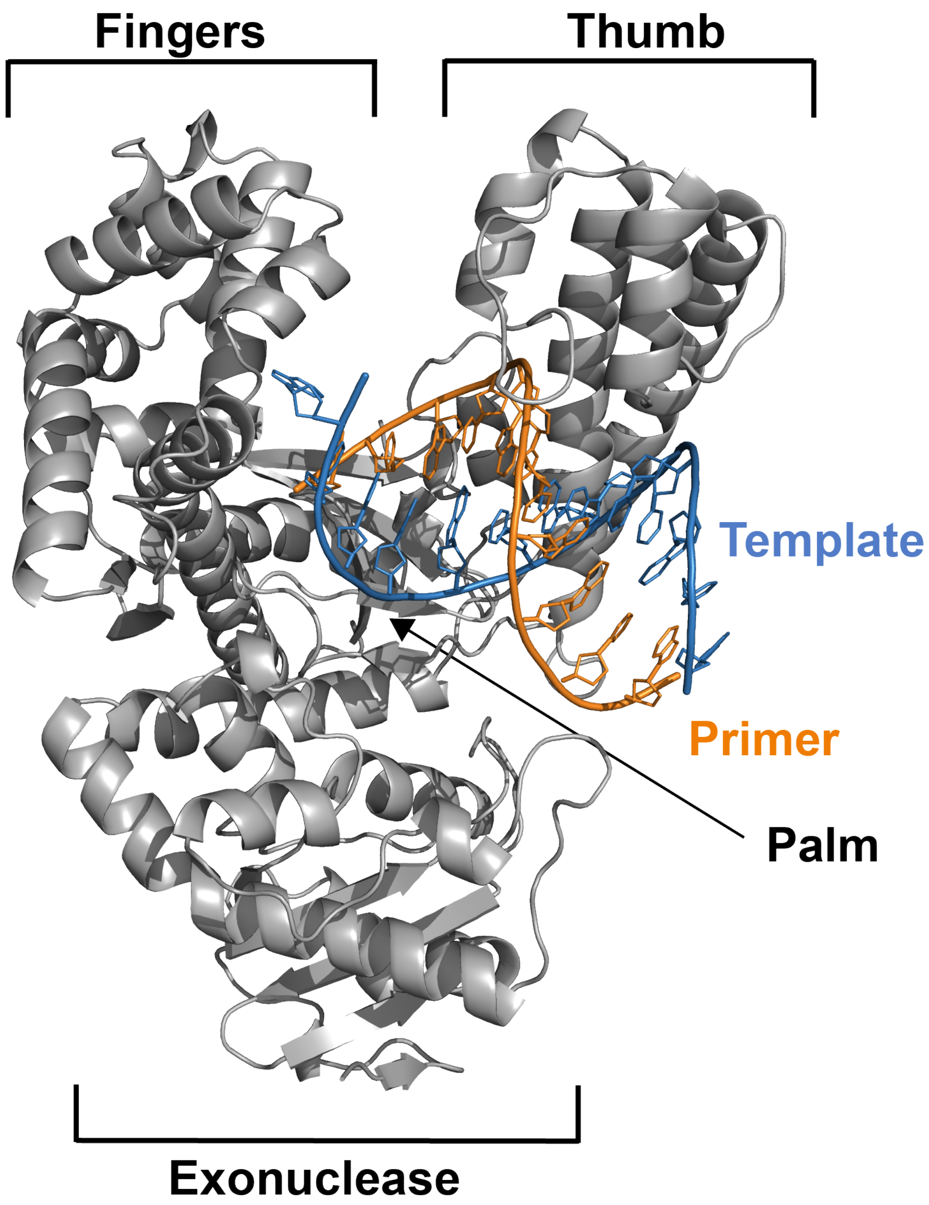

Supplementary Figure S1. Global structure of Bst DNA Polymerase bound to the DNA/FANA primer-template heteroduplex.

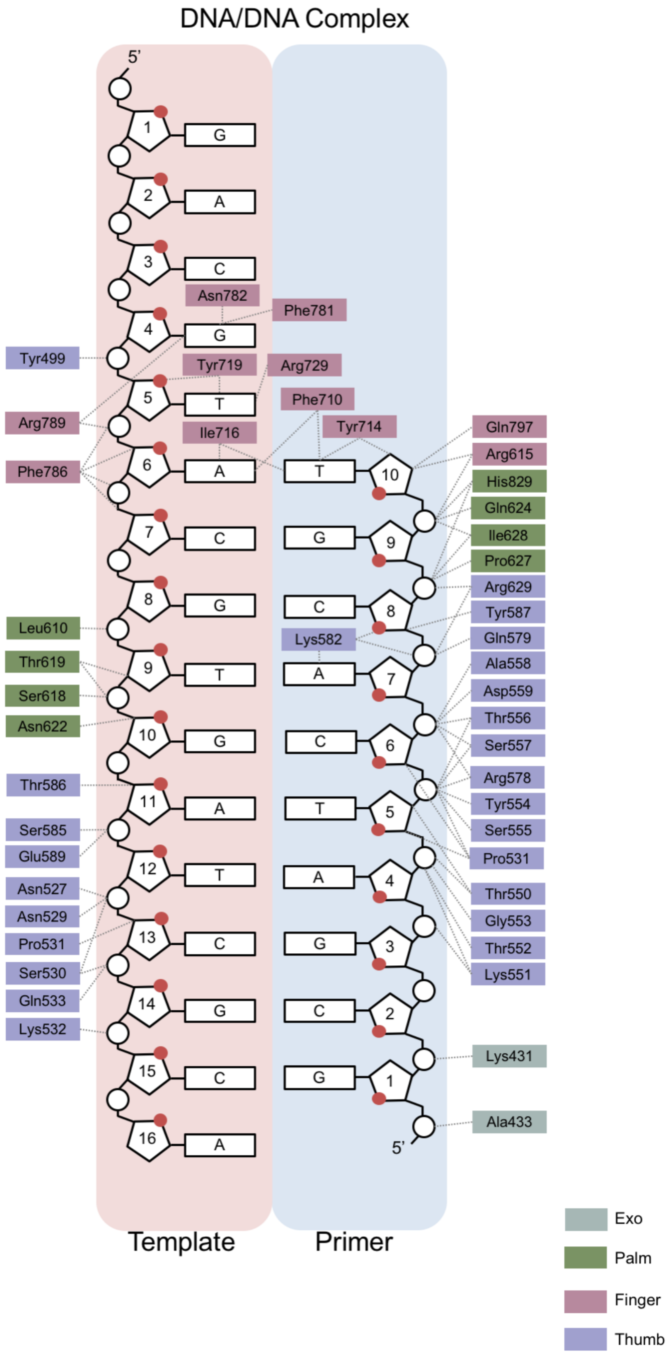

Supplementary Figure S2. Two-dimensional interaction map for the DNA binary complex. Amino acid residues are colored by polymerase subdomain. Dashed lines represent interactions between the polymerase and the duplex. Red dots represent O4' atoms in the sugar ring. This structure (PDB: 6DSY) is provided for comparison with the new structures.

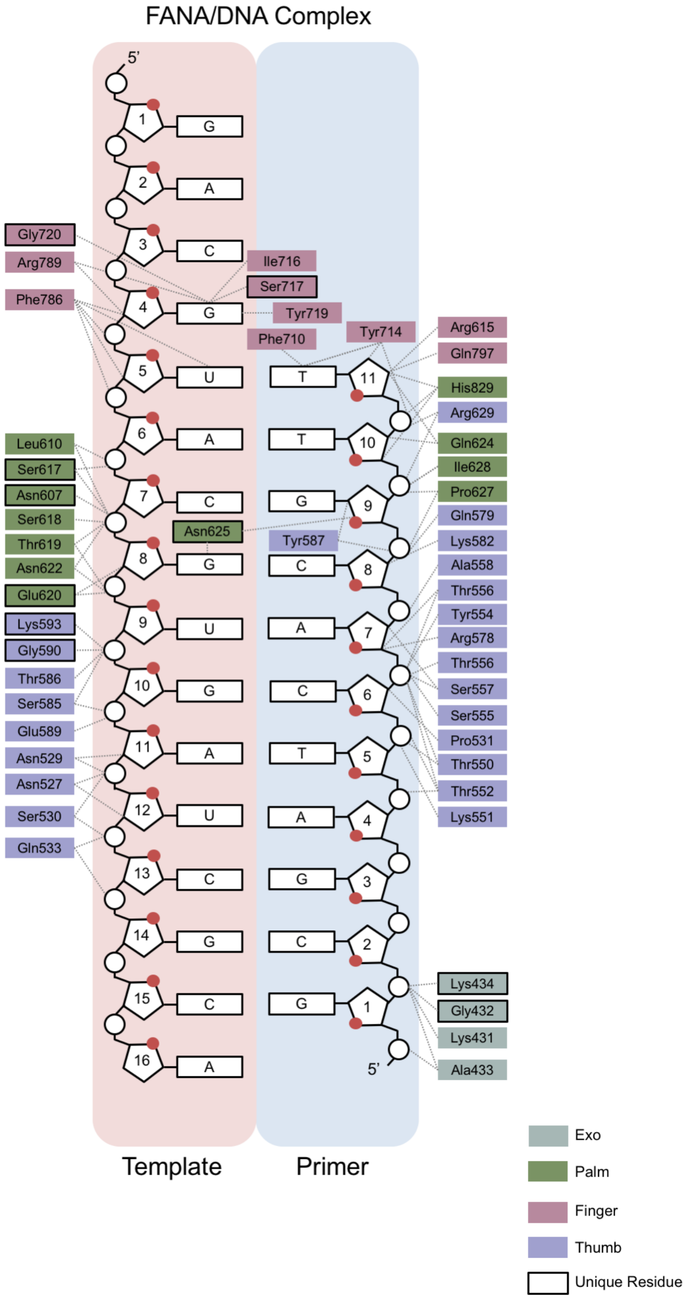

Supplementary Figure S3. Two-dimensional interaction map for the FANA binary complex. Amino acid residues are colored by polymerase subdomain and boxed residues denote interactions unique to the structure relative to the natural system. Dashed lines represent interactions between the polymerase and the primer/template duplex. Red dots represent O4' atoms in the sugar ring.

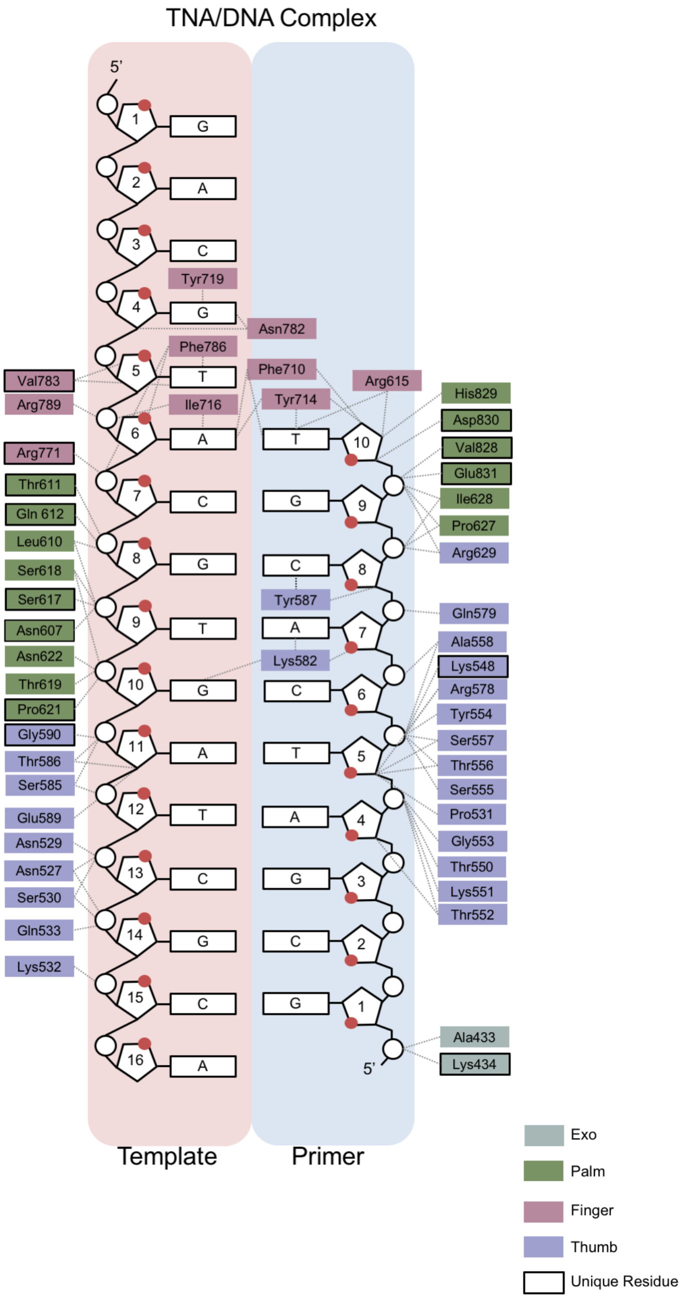

Supplementary Figure S4. Two-dimensional interaction map for the TNA binary complex. Amino acid residues are colored by polymerase subdomain and boxed residues denote interactions unique to the structure relative to the natural system. Dashed lines represent interactions between the polymerase and the primer/template duplex. Red dots represent O4' atoms in the sugar ring.

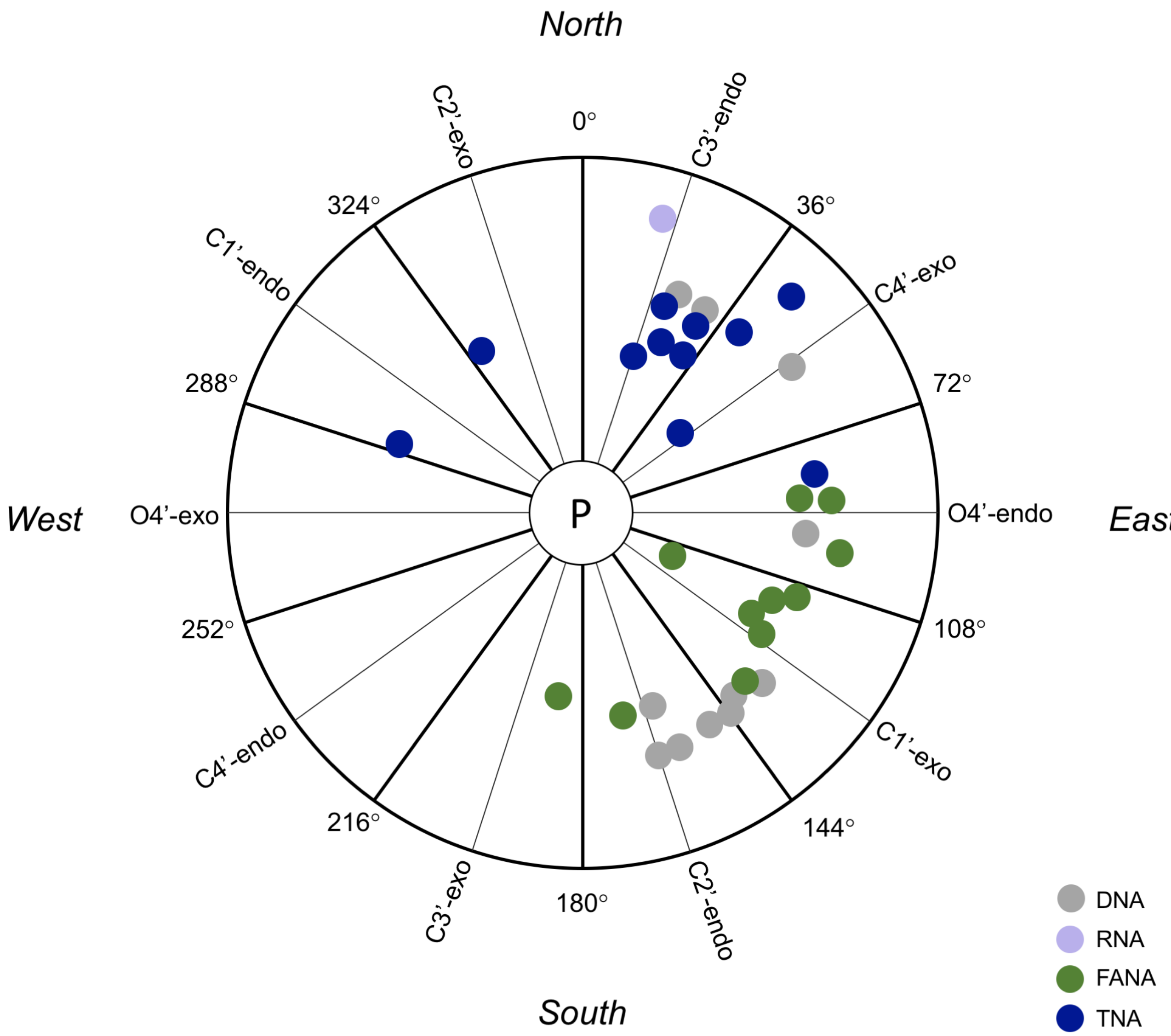

Supplementary Figure S5. Pseudorotational phase angles for Bst DNA Polymerase templates. PROSIT was used to calculate the phase angles for each duplex, including the natural system (PDB: 6DSY). Phase angles for template strand residues from each structure were plotted against the maximum dihedral angle for the residue (v_{max}) to provide information about the sugar pucker of each templating residue. Terminal residues from each duplex were excluded and a representative spot was included for a standard RNA C3'-endo sugar pucker (PDB: 3ND4).

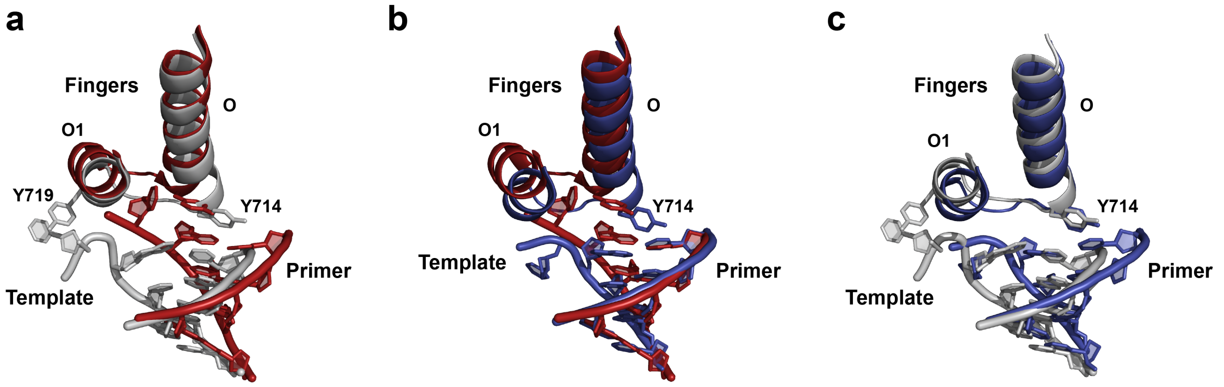

Supplementary Figure S6. Crystal structures of post-translocated Bst DNA polymerase. (a) Structural comparison showing two different conformations obtained for the binary structure of Bst DNA polymerase bound to the all-natural DNA primer-template duplex. PBD: 6DSY (grey) versus 1L3T (red). The two different conformations, which signify different steps in the reaction pathway, were obtained using different crystallization approaches. (b-c) Structural overlay of the new TNA containing binary structure (blue) against both previously solved conformations obtained for Bst DNA polymerase bound to the natural DNA template.

Supplementary Table S1. Bst DNA Polymerase complex helical (base stacking) statistics.

Primer (DNA)	DNA	FANA	TNA
**dG1**	**dC15**	**fC15**	**tC15**
Shift (Å)	-0.73	-0.79	-0.32
Slide (Å)	-1.06	-0.42	0.01
Rise (Å)	3.07	3.06	3.24
Tilt (°)	-4.75	-1.54	-4.22
Roll (°)	5.08	-2.91	1.08
Twist (°)	31.91	33.76	31.90
Inclination (°)	9.10	-4.99	1.96
**dC2**	**dG14**	**fG14**	**tG14**
Shift (Å)	0.18	0.07	-0.64
Slide (Å)	0.16	-0.13	-0.24
Rise (Å)	3.65	2.84	3.18
Tilt (°)	7.32	-8.62	2.49
Roll (°)	0.22	9.25	8.83
Twist (°)	41.34	32.04	27.82
Inclination (°)	0.31	16.00	17.76
**dG3**	**dC13**	**fC13**	**tC13**
Shift (Å)	-0.48	-0.39	-0.23
Slide (Å)	-0.02	-0.59	-0.47
Rise (Å)	3.33	3.23	3.21
Tilt (°)	-3.43	0.17	-4.58
Roll (°)	4.97	-0.94	1.87
Twist (°)	34.37	33.09	37.33
Inclination (°)	8.33	-1.65	2.91
**dA4**	**dT12**	**fU12**	**tT12**
Shift (Å)	-0.45	0.65	-0.06
Slide (Å)	-0.63	-0.88	-0.57
Rise (Å)	3.29	3.53	3.11
Tilt (°)	-2.25	6.09	1.32
Roll (°)	4.13	6.29	4.96
Twist (°)	29.14	31.23	24.73
Inclination (°)	8.15	11.41	11.43
**dT5**	**dA11**	**fA11**	**tA11**
Shift (Å)	0.94	-0.76	-0.01
Slide (Å)	-0.64	-0.76	-0.14
Rise (Å)	3.12	3.28	3.15
Tilt (°)	4.42	-5.77	0.11
Roll (°)	5.04	5.50	3.86
Twist (°)	25.18	33.04	35.41
Inclination (°)	11.31	9.51	6.32
**dC6**	**dG10**	**fG10**	**tG10**
Shift (Å)	0.20	0.38	-0.72
Slide (Å)	0.34	-0.56	-0.55
Rise (Å)	3.32	3.04	3.46
Tilt (°)	-5.27	-0.66	-1.95
Roll (°)	13.93	5.83	16.00
Twist (°)	44.47	28.73	28.30
Inclination (°)	17.82	11.60	29.86
**dA7**	**dT9**	**fU9**	**tT9**
Shift (Å)	-0.07	-0.41	-1.00
Slide (Å)	-1.23	-0.70	-0.41
Rise (Å)	3.34	3.38	3.58
Tilt (°)	-0.99	4.15	-7.21
Roll (°)	-2.40	9.07	3.50
Twist (°)	27.42	29.41	34.74
Inclination (°)	-5.05	17.26	5.77
**dC8**	**dG8**	**fG8**	**tG8**
Shift (Å)	0.45	-0.01	-0.63
Slide (Å)	-0.50	-0.27	0.19
Rise (Å)	3.78	3.06	3.18
Tilt (°)	9.84	-3.67	2.01
Roll (°)	10.16	7.96	9.61
Twist (°)	44.94	33.03	30.13
Inclination (°)	12.92	13.70	17.90
**dG9**	**dC7**	**fC7**	**tC7**
Shift (Å)	0.72	0.23	0.18
Slide (Å)	-0.72	-0.91	-0.17
Rise (Å)	3.74	3.35	2.99
Tilt (°)	9.38	6.05	2.88

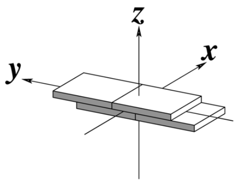

Slide

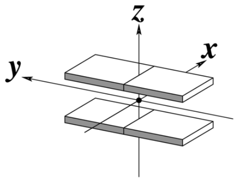

Rise

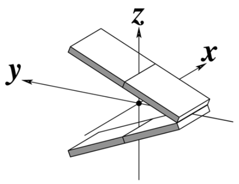

Tilt

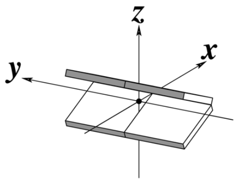

Roll

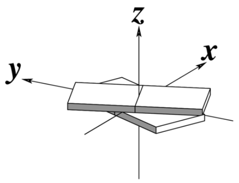

Twist

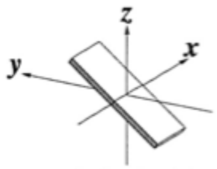

Inclination

Roll (°)	-1.14	1.26	0.26
Twist (°)	29.52	32.91	24.10
Inclination (°)	-2.16	2.20	0.61
**dT10**	**dA6**	**fA6**	**tA6**
Shift (Å)		0.46	
Slide (Å)		-0.14	
Rise (Å)		3.06	
Tilt (°)		2.75	
Roll (°)		3.73	
Twist (°)		43.71	
Inclination (°)		4.99	

Supplementary Table S2. Bst DNA Polymerase complex base pair statistics.

Primer (DNA)	DNA	FANA	TNA
**dG1**	**dC15**	**fC15**	**tC15**
Shear (Å)	0.70	-1.06	-1.05
Stretch (Å)	0.28	-0.58	-0.41
Stagger (Å)	0.44	-0.24	-0.15
Buckle (°)	4.01	-18.00	-11.68
Propeller (°)	-5.19	-13.67	-7.54
Opening (°)	-0.28	-5.44	-4.93
**dC2**	**dG14**	**fG14**	**tG14**
Shear (Å)	-0.42	0.15	0.42
Stretch (Å)	-0.62	0.01	-0.17
Stagger (Å)	0.83	-0.10	0.27
Buckle (°)	11.40	-7.03	-8.77
Propeller (°)	-20.11	-10.98	-11.29
Opening (°)	-5.02	-4.25	6.07
**dG3**	**dC13**	**fC13**	**tC13**
Shear (Å)	0.07	-0.15	-0.30
Stretch (Å)	0.42	-0.09	-0.24
Stagger (Å)	-0.01	0.77	-0.13
Buckle (°)	-0.53	5.83	-3.90
Propeller (°)	-18.76	-9.77	-9.99
Opening (°)	9.82	2.05	2.32
**dA4**	**dT12**	**fU12**	**tT12**
Shear (Å)	0.75	-0.21	0.35
Stretch (Å)	-0.06	-0.33	-0.08
Stagger (Å)	0.14	0.47	0.08
Buckle (°)	-7.14	8.04	-1.82
Propeller (°)	-18.93	-14.84	-14.68
Opening (°)	4.93	0.16	1.58
**dT5**	**dA11**	**fA11**	**tA11**
Shear (Å)	-0.06	0.05	-0.52
Stretch (Å)	-0.13	-0.04	-0.15
Stagger (Å)	0.17	-0.02	-0.01
Buckle (°)	-3.18	-1.46	2.22
Propeller (°)	-7.63	-17.52	-10.93
Opening (°)	-3.74	11.53	9.32
**dC6**	**dG10**	**fG10**	**tG10**
Shear (Å)	-1.56	0.14	0.03
Stretch (Å)	-0.12	0.20	-0.00
Stagger (Å)	-0.16	0.30	-0.23
Buckle (°)	2.74	-2.70	8.28
Propeller (°)	-8.31	-6.05	-10.54
Opening (°)	-0.05	-3.20	2.58
**dA7**	**dT9**	**fU9**	**tT9**
Shear (Å)	1.38	-0.20	0.25
Stretch (Å)	-0.27	0.03	-0.19
Stagger (Å)	0.54	0.40	-0.10
Buckle (°)	9.46	2.65	5.97
Propeller (°)	-6.78	-3.46	-8.83
Opening (°)	3.73	3.02	-1.43
**dC8**	**dG8**	**fG8**	**tG8**
Shear (Å)	-0.39	0.34	0.12
Stretch (Å)	-0.39	-0.14	-0.07
Stagger (Å)	0.39	0.21	0.40
Buckle (°)	3.02	3.48	-7.67
Propeller (°)	-13.44	-5.19	-17.46
Opening (°)	1.45	-0.83	1.94
**dG9**	**dC7**	**fC7**	**tC7**
Shear (Å)	1.07	-0.41	-0.20
Stretch (Å)	-0.06	-0.12	-0.17
Stagger (Å)	-0.39	0.61	0.14
Buckle (°)	-18.72	-0.58	3.99
Propeller (°)	-17.77	-23.42	-3.37
Opening (°)	-1.67	8.27	-5.35
**dT10**	**dA6**	**fA6**	**tA6**
Shear (Å)	-1.15	-0.05	-0.33
Stretch (Å)	-1.08	0.05	-0.06
Stagger (Å)	-0.84	0.24	-0.21
Buckle (°)	-26.72	-5.30	19.75
Propeller (°)	-19.00	-20.51	-3.03
Opening (°)	6.14	7.77	1.16

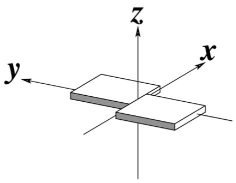

Shear

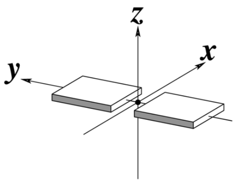

Stretch

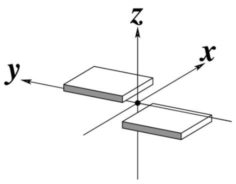

Stagger

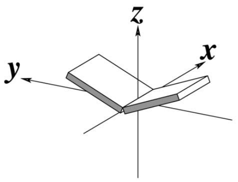

Buckle

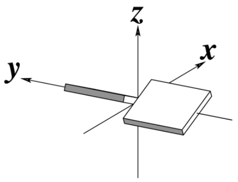

Propeller

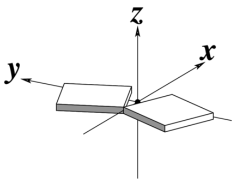

Opening

dT11	dT5	fU5	tT5
Shear (Å)		2.38	
Stretch (Å)		-1.72	
Stagger (Å)		0.46	
Buckle (°)		10.03	
Propeller (°)		-18.39	
Opening (°)		10.20	

Supplementary Table S3. TNA torsion angles

	B-TNA*	A-TNA**
	**tA6**	**tC1**
α (°)	65.7	
γ (°)	-119.5	
δ (°)	150.1	144.1
ε (°)	-138.4	-40.8
ζ (°)	-70.6	-149.6
	**tC7**	**tG2**
α (°)	-94.3	-76.4
γ (°)	-177.2	157.6
δ (°)	158.6	115.4
ε (°)	-98.3	-125.2
ζ (°)	-85.5	-74.8
	**tG8**	**tA3**
α (°)	-91.0	-80.1
γ (°)	163.3	-176.5
δ (°)	159.3	151.0
ε (°)	-110.7	-106.7
ζ (°)	-75.3	-101.0
	**tT9**	**tA4**
α (°)	-81.8	-79.1
γ (°)	168.9	-178.3
δ (°)	154.5	157.9
ε (°)	-97.0	-120.0
ζ (°)	-74.0	-83.2
	**tG10**	**tT5**
α (°)	-91.0	-82.5
γ (°)	167.9	-166.6
δ (°)	150.5	153.3
ε (°)	-98.2	-89.7
ζ (°)	-92.6	-84.2
	**tA11**	**tT6**
α (°)	-82.8	-56.3
γ (°)	153.6	-180.0
δ (°)	146.6	109.2
ε (°)	-79.5	-96.3
ζ (°)	-97.7	-81.7
	**tT12**	**tC7**
α (°)	-83.4	-79.8
γ (°)	155.0	177.9
δ (°)	147.3	146.8
ε (°)	-120.7	-104.1
ζ (°)	-64.6	-70.8
	**tC13**	**tG8**
α (°)	-94.6	-130.0
γ (°)	-156.2	172.5
δ (°)	132.6	
ε (°)	166.7	
ζ (°)	-42.2	
	**tG14**	
α (°)	148.0	
γ (°)	-63.4	
δ (°)	139.1	
ε (°)	61.9	
ζ (°)	37.5	
	**tC15**	
α (°)	109.9	
γ (°)	-38.6	
δ (°)	136.3	
ε (°)	59.1	
ζ (°)	81.4	

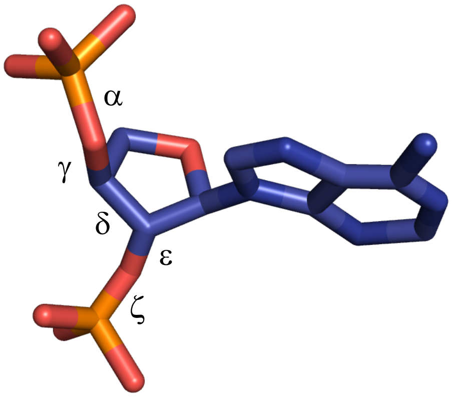

*Values, from this work, reporting TNA template parameters of residues that form a duplex with the DNA primer. Torsion angle nomenclature based on Ref 1.
**Values derived from self-complementary 8-mer strand from Ref 2.

1. Wilds, C.J., Wawrzak, Z., Krishnamurthy, R., Eschenmoser, A. and Egli, M. (2002) Crystal structure of a B-form DNA duplex containing (L)-a-threofuranosyl (3'-2') nucleosides: a four-carbon sugar is easily accommodated into the backbone of DNA. *J. Am. Chem. Soc.*, **124**, 13716-13721.
2. Ebert, M.-O., Mang, C., Krishnamurthy, R., Eschenmoser, A. and Jaun, B. (2008) The structure of a TNA-TNA complex in solution: NMR study of the octamer duplex derived from a-(L)-threofuranosyl-(3'-2')-CGAATTCG. *J. Am. Chem. Soc.*, **130**, 15105-15115.

Supplementary Table S4. Bst DNA Polymerase complex sugar puckers.

T – P	DNA System Template (DNA)	DNA System Primer (DNA)	FANA System Template (FANA)	FANA System Primer (DNA)	TNA System Template (TNA)	TNA System Primer (DNA)
U5 – T11	--	--	C1’-exo	C3’-endo	--	--
A6 – T10	C2’-endo	C3’-endo	C2’-endo	C3’-endo	C1’-endo	C1’-exo
C7 – G9	C2’-endo	C3’-endo	C1’-exo	C3’-endo	C3’-endo	C2’-endo
G8 – C8	C3’-endo	C4’-exo	C3’-exo	C2’-endo	C3’-endo	C4’-exo
T9 – A7	O4’-endo	C2’-endo	O4’-endo	C1’-exo	C4’-exo	C2’-endo
G10 – C6	C1’-exo	C1’-exo	C1’-exo	C1’-exo	C3’-endo	C1’-exo
A11 – T5	C2’-endo	C1’-exo	O4’-endo	C3’-exo	C3’-endo	C1’-exo
T12 – A4	C1’-exo	C2’-endo	C1’-exo	C2’-endo	C3’-endo	C1’-exo
C13 – G3	C1’-exo	C3’-exo	O4’-endo	C2’-endo	C1’-endo	C2’-endo
G14 – C2	C4’-exo	C3’-exo	C1’-exo	C1’-exo	C4’-exo	C3’-endo
C15 – G1	C2’-endo	C2’-endo	C1’-exo	C3’-exo	O4’-endo	C3’-exo
**Average**	**C2’-endo**	**C2’-endo**	**C1’-exo**	**C1’-exo**	**C3’-endo**	**C1’-exo**

Supplementary Table S5. Bst DNA Polymerase complex intrastrand P_i – P_{i+1} distances (Å).

T – P	DNA System Template (DNA)	DNA System Primer (DNA)	FANA System Template (FANA)	FANA System Primer (DNA)	TNA System Template (TNA)	TNA System Primer (DNA)
U5 – T11	--	--	6.1	6.4	--	--
A6 – T10	6.8	6.6	6.2	7.0	5.4	6.6
C7 – G9	7.5	6.6	7.1	6.4	5.8	6.2
G8 – C8	7.0	6.7	6.5	6.6	5.9	6.5
T9 – A7	7.0	6.7	6.8	6.6	5.8	6.8
G10 – C6	6.8	6.5	6.4	6.8	5.9	6.5
A11 – T5	6.4	6.4	6.7	6.6	5.6	6.5
T12 – A4	6.6	7.5	6.5	6.8	5.9	6.9
C13 – G3	6.7	6.9	6.5	6.7	6.0	6.9
G14 – C2	6.8	6.1	6.7	7.0	5.3	7.4
C15 – G1	6.9	--	6.7	--	5.3	--
**Average**	**6.85**	**6.67**	**6.56**	**6.69**	**5.69**	**6.7**
